# Functional outcome of fusion versus ligament reconstruction in patients with a syndesmosis injury: A narrative review

**DOI:** 10.1016/j.asmart.2021.05.002

**Published:** 2021-06-12

**Authors:** Sai-Kit Lim, Yui-Chung Ho, Samuel Ka-Kin Ling, Patrick Shu-Hang Yung

**Affiliations:** Department of Orthopaedics and Traumatology, Faculty of Medicine, The Chinese University of Hong Kong (CUHK), Hong Kong

**Keywords:** Ankle, AITFL, Syndesmosis, Arthrodesis, Fusion, Ligament reconstruction

## Abstract

Injury to distal tibiofibular syndesmosis is commonly associated with ankle fractures. The surgical treatment especially in isolated chronic syndesmosis instability is still debated. This article has reviewed literature identified from PubMed, EMBASE and Cochrane from year 2000 onwards and compared the functional outcomes between syndesmosis fusion and ligament reconstruction based on Preferred Reporting Items for Systematic Reviews and Meta-Analyses (PRISMA) guidelines. Eighteen studies were included. All the included papers described a good-to-excellent post-operative functional outcome without major complications. No significant difference between the two surgical interventions could be concluded. Further studies of better quality shall be conducted in the future.

## Introduction

The distal tibiofibular syndesmotic complex is a major contributor to the dynamic stability of the ankle joint. The structure composes of the anterior-inferior tibiofibular ligament (AITFL), the posterior-inferior tibiofibular ligament (PITFL), and the interosseous ligament (IOL). Disruption of the tibiofibular syndesmotic complex may lead to ankle instability and asymmetrical mortise.^1^

Syndesmosis injuries are often associated with ankle fractures. The classical presentation is pronation-external rotation ankle fracture according to the Lauge-Hansen classification,^2^ but the AITFL rupture caused by the supination-external rotation mechanism was also common.^3^ Isolated syndesmotic disruption is rare, which only accounts for 1–11% of total syndesmosis injury.^4^ Under-diagnosis or malreduction may result in chronic instability and joint degeneration.^5^

Syndesmosis injury can be diagnosed with clinical examination and radiological imaging. Common manual testing methods such as cotton test and syndesmotic stress test. A positive diagnosis is defined as significant syndesmotic diastasis over 6 mm or 44% fibular width.^6^ The tibiofibular clear space (TCS), the medial clear space (MCS), and the tibiofibular overlapping (TFO) are compared bilaterally to rule out tibiofibular malunion. However, plain radiographic assessment and intra-operative fluoroscopic assessment may be inadequate^7^; therefore, computer tomography (CT) and magnetic resonance imaging (MRI) analysis are recommended.^1^^,^^7^^,^^34^ Despite the effectiveness of manual tests and radiographic techniques in identifying syndesmotic injuries, the test results have no proven efficacy to guide between which surgical option is more suitable.^4^

Rigid stabilization with screws or suture-like elastic constructs are often used to stabilize the affected structure, however, high rates of malunion (up to 50%)^8^, ^9^, ^10^, ^11^ and complications such as broken implant are reported. Reconstruction of the ruptured ligament, i.e. especially the AITFL, is an alternative method to regain ankle integrity. Currently, there was no consensus on the operative procedure or the choice of donor graft tendon for reconstruction. The majority of studies on syndesmosis repair currently available are cohort studies without comparison groups, thus it is difficult to conclude on an ideal technique without more in-depth evidence. This review aims to compare the functional outcome and complications between these techniques and report the up-to-date findings on the treatment of the syndesmotic injury.

## Materials and methods

### Data source

The narrative review used the Preferred Reporting Items for Systematic Reviews and Meta-Analyses (PRISMA) guidelines as reference^12^. Major medical databases: PubMed, EMBASE and Cochrane were searched on June 1, 2020, for studies from 2000 to 2020 with the following search strategy: (1) AND (2) OR (1) AND (3) Table 1.

**Table 1:** Search terms used for literature search on June 1, 2020.

Group	Search terms
1	“syndesmosis” or “syndesmotic” or “high ankle” or “anterior inferior tibiofibular ligament” or “posterior inferior tibiofibular ligament” or “AITFL^a^” or “PITFL^b^” or “deltoid ligament” or “tibiofibular” or “tibiofibular diastasis”
2	“fusion∗” or “arthrodesis” or “screw” or “fixation” or “plate” or “suture” or “button” or “tightrope” or “endobutton∗” or “implant∗” or “stabilize” or “stabilization∗”
3	“reconstruction” or “reconstruct” or “ligament∗” or “ligamentous” or “ligamentoplasty” or “repair”

a AITFL, anterior-inferior tibiofibular ligament.

b PITFL, posterior-inferior tibiofibular ligament.

### Study selection

**Fig. 1:**
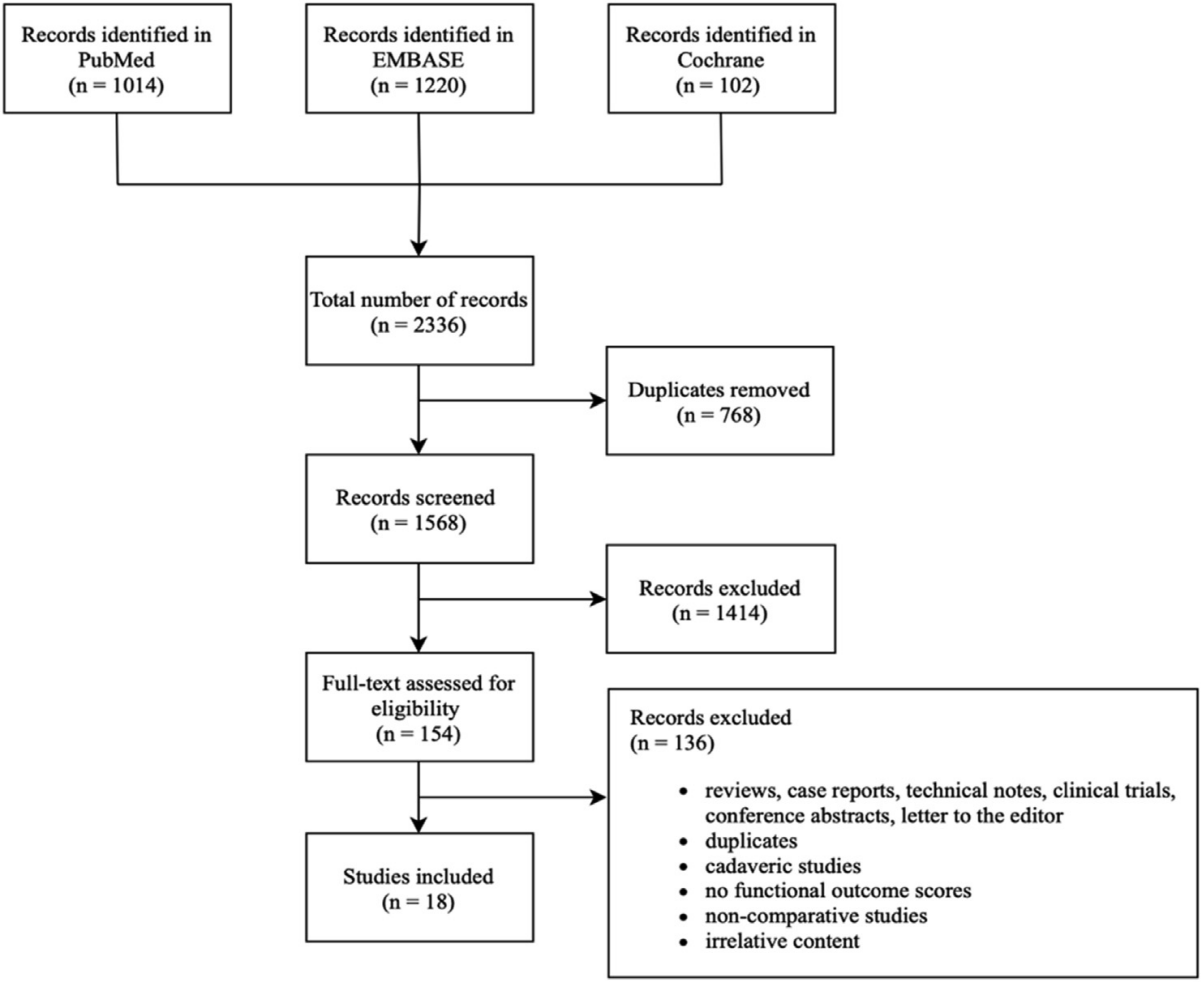
A flow chart illustrating the process of literature search and selection according to the guidelines of PRISMA.

Studies were included if they were original research (excluding cadaveric studies) that assess the clinical outcome of patients with surgically treated syndesmotic injuries. Studies were excluded if they were not human studies, not written in English, failed to provide full text, lack complete data (i.e., without functional outcome scores), or published as case reports, technical reports, clinical trials or review articles (Fig. 1). The review and selection process was carried out by two reviewers S.K.L and Y.C.H. independently. Conflicts were solved by discussion and mutual agreement.

### Data extraction

The following data were extracted from the included studies: the number of patients and ankle treated, age, follow-up time, study designs, detection methods, surgical technique, clinical outcomes (pre-and postoperative), and complications. Studies were separated into two groups: fusion of tibiofibular joint and ligament reconstruction.

### Study quality assessment

The quality and risk of bias of included studies were assessed using the Methodological Index for Nonrandomized Studies (MINORS) tool.^13^ Each study (both comparative and non-comparative) was evaluated for the aim of study, inclusion of consecutive patients, prospective collection of data, evaluation of endpoint, and follow-up rate and period. For comparative studies, additional items of control group, baseline equivalence, prospective calculation of study size, and statistical analysis of study design were evaluated. The ideal score for non-comparative studies was 16 and for comparative studies were 24.

## Results

### Included studies

The search strategy had identified 2336 potential studies. After removing 768 duplicates, 1568 studies have proceeded to title and abstract screening. Based on the mentioned inclusion and exclusion criteria, 1414 studies were excluded, and 154 studies were subjected to detailed full-text screening. 18 outcome studies were included at the end of the screening and separated into two groups as shown in Fig. 1.

### Study characteristics

There were 836 patients (836 ankles) treated. Two (2) studies were carried out as randomized controlled trials while others were either cohort studies or case series.^7^^,^^9^ Among all the cohort studies and case series studies, only two of them the data were collected prospectively. Only six studies were comparative while others had no comparison group (see Table 2).

**Table 2:** Evaluation of the risk of bias using MINORS tool.

Study	Year	Study Design	Pros/Retro	Comparison group	MINORS score^a^
Kocadal et al.^16^	2016	Cohort	Retrospective	Yes	17
Rigby et al.^17^	2013	Cohort	Retrospective	No	11
Pakarinen et al.^3^	2011	Cohort	Retrospective	Yes	16
Wikerøy et al.^9^	2010	Randomized trial	Prospective	Yes	20
De Vil et al.^18^	2009	Cohort	Retrospective	No	11
Kortekangas et al.^7^	2015	Randomized trial	Prospective	Yes	23
Kwaadu et al.^19^	2015	Case series	Retrospective	No	11
Grass et al.^20^	2003	Cohort	Retrospective	No	10
Cottom et al.^14^	2009	Cohort	Prospective	Yes	14
Wagener^5^	2011	Case series	Prospective	No	10
Schuberth et al.^21^	2008	Case series	Retrospective	No	10
Olson et al.^22^	2011	Case series	Retrospective	No	11
Yausui et al.^23^	2010	Case series	Retrospective	No	7
Morris et al.^24^	2009	Case series	Retrospective	No	8
Steinmetz et al.^6^	2016	Cohort	Retrospective	No	10
Seyhan et al.^25^	2015	Cohort	Retrospective	Yes	17
Colcuc et al.^4^	2016	Cohort	Retrospective	No	8
Zamzami & Zamzam^1^	2006	Cohort	Retrospective	No	9

a MINORS socre, Methodological Index for Nonrandomized Studies score.

### Surgical treatment

The most commonly reported mechanism of injury was pronation-external rotation injury associated with an ankle fracture.^3^ The diagnosis of syndesmotic injury and the decision for surgical intervention was decided by clinical examination (such as squeeze test or external rotation test), presence of symptoms, or abnormal ankle radiographs. A total of 337 patients underwent fixation of the tibiofibular joint screws and tightropes were the most common choice of the fixation device Table 3. Out of 10 studies in the fusion group, three studies used only screws while only one study used Tightrope as the sole fixation device. Four (4) studies had combined the use of screws and Tightropes. A bolt was chosen to stabilize the tibiofibular joint in one study. AITFL reconstruction was performed on 499 patients Table 4. Among the 8 studies included, tendon transfer grafts included split peroneus longus, plantaris, semitendinosus, gracillis and hamstring had been adapted in 5 studies respectively. Plantaris tendon reconstruction was used to treat patients with AITFL rupture in one study.^4^ One had reconstructed the PITFL in addition to the AITFL using an ipsilateral semitendinosus tendon. The mean follow-up time for the fusion group was 35.7 months (range 4–139 months) while that for the ligament reconstruction group was 43.5 months (range 6–126 months). Overall follow-up time was 40.6 months.

**Table 3:** Summary of individual studies that prescribed tibiofibular fixation as the surgical intervention.

Study	Patients (ankles)	Mean Age (SD^a^ or range)	Surgical technique	Complications	Mean follow-up	Scoring system	Mean Pre-operation functional score	Mean Post-operation functional score (SD^a^ or range)
Kocadal et al.^16^	52 Screw (26) Suture-button (26)	44.1 ± 13.2 (16–65) Screw 44.8 ± 11.3 (16–65) Suture-button 43.4 ± 15.1 (16–61)	Screw fixation with 3.5 mm cortical screw, 4 cortices Suture-button fixation	1 low-grade infection 3 transient tightness in dorsiflexion 1 implant irritation	16.7 ± 11 months (6-43)	AOFAS^c^	NR^b^	86.1 ± 14.0 Screw fixation 88.4 ± 9.2 Suture button fixation
Rigby et al.^17^	37 (37)	40.7 ± 18.0	Single or double TightRope® (Arthrex, Inc., Naples, FL, USA)	7 knot irritation	23.6 ± 4.3 months	AOFAS^c^	NR^b^	97(90–100)
Wikerøy et al.^9^	48 Group1 (23) Group2 (25)	45.9 (14.9) Group 1 52.4 (15.1) Group 2	Fixation with one quadricortical screw for group 1 and two tricortical screws for group 2	NR^b^	8.4 years	OMA^d^ OTA^e^	NR^b^	82.8 (19.9) OMA Group 1 82.3 (19.4) OMA Group 2 84.3 (13.3) OTA Group 1 88.5 (11.6) OTA Group 2
De Vil et al.^18^	28(28)	44 (16–65)	Bolt fixation	5 skin irritations	66 (24–139) months	AOFAS^c^	NR^b^	86 (33–100)
Kortekangas et al.^7^	43 Tightrope® (21) Screw (22)	46.0 (14.8) Tightrope® 43.5 (15.7) Screw	Fracture fixation followed by syndesmotic screw fixation with one 3.5-mm cortical screw purchasing three cortices or with one TightRope®	1 infection 3 broken screw 13 loosened screw	At least 2 years 36 months Tightrope® 37 months Screw	OMA^d^	NR^b^	82 Tightrope® 84 Screw
Kwaadu et al.^19^	31 (31)	48.4 (27–84)	Lagged screw fixation	1 irritation	18 (10–46) months	AOFAS^c^	NR^b^	88.4 (42–100)
Cottom et al.^14^	50 Tightrope® (25) Screw (25)	34.68 (15–55) Tightrope® 36.68 (17–74) Screw	Transosseous fixation using screw or Tightrope®	NR^b^	10.78 (6–12) months Tightrope® 8.2 (4–24) months Screw	Modified AOFAS^c^ with maximum score of 63	29.84 (0–35) Tightrope® 33.42 (0–40) Screw	50.64 (30–63) Tightrope® 53.45 (25–63) Screw
Schuberth et al.^21^	6 (6)	47 (29–62)	Arthroscopic assisted debridement followed by *trans*-syndesmotic screw fixation	1 tibiofibular synostosis	32 (24–64) months	AOFAS^c^	56.3 (52–67)	88.7 (79–100)
Olson et al.^22^	10 (10)	44 (40–63)	Syndesmosis arthrodesis with placement of cancellous bone graft in between distal tibial fibular space	NR^b^	41 (29–44) months	AOFAS^c^	37 ± 15 (16–62)	87 ± 11 (70–100)
Seyhan et al.^25^	32 Screw (17) Elastic fixation (15)	32.0 Screw 33.2 Elastic fixation	4 cortex single cortical screw fixation for screw group. Single level TightRope® fixation for elastic fixation group.	No complication	12 months	AOFAS^c^	NR^b^	93.35 ± 6.93 Screw 93.73 ± 7.38 Elastic fixation

a SD, standard deviation.

b NR, not reported.

c AOFAS, American Orthopaedic Foot and Ankle Society.

d OMA, Olerud Molander Ankle.

e OTA, Orthopaedic Traumatic Association.

**Table 4:** Summary of individual studies that prescribed ligament reconstruction as the surgical intervention.

Study	Patients (ankles)	Mean Age (SD^a^ or range)	Surgical technique	Complications	Mean follow-up (range)	Scoring system	Mean Pre-operation score	Mean Post-operation score (SD^a^ or range)
Pakarinen et al.^2^	288 Group1 (165) Group2 (123)	47.7 (15–81) Group 1 47.5 (13–88) Group 2	Screw fixation of fracture with AITFL^e^ repaired in group 1 and not repaired in group 2	NR^b^	36 (24–48) Months Group 1 39 (29–70) Months Group 2	OMA^d^	NR^b^	77(25) Group 1 73(26) Group 2
Grass et al.^20^	16(16)	40	Tibiofibular syndesmosis reconstruction for chronic instability using split peroneus longus tendon	1 broken screw	16.4 (13–29) months	Karlsson Ankle Functional Score	NR^b^	88 (70–100)
Wagener et al.^5^	12(12)	32 (17–54)	Reconstruction of ATFL^f^ by creating a bone block on tibia with syndesmosis fixation by 4 cortices screw	No complication	25 (6–51) months	AOFAS^c^	72 (59–85)	92 (76–100)
Yausui et al.^23^	6(6)	23 (19–56)	Reconstruction of AITFL^e^ with autogenous gracilis tendon and interference screw	No complication	38 (31–50) months	AOFAS^c^	NR^b^	95 (90–100)
Morris et al.^24^	8(8)	32 (17–46)	ATFL^f^ reconstruction using free hamstring autograft	1 infection	39 (9–86) months	AOFAS^c^ Maryland Foot Score	NR^b^	AOFAS^c^ 85.4 (49–100) Maryland 89.3 (63–100)
Steinmetz et al.^6^	126(126)	45 ± 15.7	ATFL^f^ repair through bone tunnel with absorbable suture and screw fixation with three or four cortices. Double fixation.	5 infections 2 ankle stiffness 12 complex regional pain syndrome 1 deep venous thrombosis	5.9 ± 5.7 (2.9–10.5) years	AOFAS^c^ OMA^d^	NR^b^	AOFAS^c^ 93 ± 9 (49–100) OMA^d^ 93 ± 10(45–100)
Colcuc et al.^4^	32 Grade I (10) Grade II (12) Grade III (10)	41 (18–71)	Arthroscopically assisted stabilization tibiofibular joint using screw and TightRope® System (Arthrex, Inc., Naples, FL, USA), followed by syndesmosis reconstruction using one of the following techniques: suture of AITFL^e^ (grade I instability), periosteal flap (grade II instability) or autogenous plantaris tendon graft (grade III instability).	1 infection 2 suture granuloma	17 months	AOFAS^c^	67 ± 9 Grade I 68 ± 4 Grade II 53 ± 13 Grade III	93 ± 5 Grade I 93 ± 4 Grade II 86 ± 5 Grade III
Zamzami & Zamzam^1^	11	31.5 (19–44)	Reconstruction of AITFL^e^ and PITFL^g^ using ipsilateral semitendinosus tendon with syndesmotic screw stabilization	1 persistent tightness in dorsiflexion	3.1 (2–5) years	West Point Ankle Score System	NR^b^	95.4 (87–100)

a SD, standard deviation.

b NR, not reported.

c AOFAS, American Orthopaedic Foot and Ankle Society.

d OMA, Olerud Molander Ankle.

e AITFL, anterior-inferior tibiofibular ligament.

f ATFL, anterior tibiofibular ligament.

g PITFL, posterior-inferior tibiofibular ligament.

### Functional outcomes

The American Orthopaedic Foot and Ankle Society (AOFAS) score was the most common functional evaluation tool reported in the pooled studies (12 studies) followed by The Olerud Molander Ankle (OMA) score (4 studies). Other tools of functional evaluation including Karlsson Ankle Functional Score (KAFS), Maryland Foot Score, and West Point Ankle Score System was reported in one of the studies respectively. One study^14^ had modified the AOFAS therefore the maximum score was reduced to 63. Two (2) studies in the fusion group and three studies in the ligament construction group had reported both pre-operative and post-operative functional scores while the post-operative score was presented in all studies.

The AOFAS scored of 95–100 was regarded as excellent, 85 to 94 as good, 65 to 84 as fair, and less than 65 as poor.^5^ Improvement from poor or fair to good or excellent after treatment could be seen in all studies that reported both preoperative and postoperative scores. All studies reported only post-operative AOFAS had also achieved good to excellent outcomes. All studies employed OMA score as evaluation tool had also reported good (61%–90%) to excellent (91%–100%) post-operative outcome.^15^ General improvement could be observed in all the studies after surgery.

CT or weight-bearing plain imaging of TFCS, MCS and TFO are the most common methods of post-operation evaluation in addition to functional scoring checklist (3,5,7,9,14,16,17,18,20,21,22). One study has included talocrural angle and talar tilt in the post-operation imaging. Seven (7) studies did not report a post-operation imaging evaluation method.

### Complications

No major complication had been reported in the pooled studies and the general complication rate was low. Eight studies reported low-grade infection or implant irritation in a small number of patients. Persistent limitation of ankle dorsiflexion had been reported in three studies. The other two studies using screw as fixation material had reported broken or loosened screws in patients with no further complications. One study had reported a 15% of post-operative complication rate, of which 4.8% was serious.^6^

### Quality assessment

The quality of the included studies was overall satisfactory. Fourteen (14) out of 18 studies were retrospective studies. Small sampling size was a factor limiting the quality of studies, of which 7 out of 18 studies had less than 20 patients. The lack of pre-operative functional score in most studies was a major limitation to evaluate the effect of surgical intervention to treat the injury.

## Discussion

The objective of the narrative review is to compare the surgical outcome between fusion and ligament reconstruction on patients with syndesmosis injuries, it is therefore essential to elaborate on differences between the two surgical intervention; Syndesmosis injury covers a large spectrum of symptoms and the treatment on subacute cases (from acute to 6 months post-injury)^26^ and chronic instability may vary. Choice of treatment intervention depends on a number of variables including the concomitant injuries, the severity of injury, and the overall stability of the ankle mortise.

While chronic syndesmosis instability refers to a failure of ligament recovery 6 months post-trauma; the leading cause is the malunion of the fracture site, resulting in a malalignment of the tibiofibular structure.^27^ Failures are also associated with other factors such as obesity, while diabetes mellitus and frequent smoking are less correlated with a failure in recovery.^28^

Major finding of the studies comprises:

1. Out of 18 includes studies, only 6 of them are comparative; nevertheless, none were direct comparisons between fusion and ligament reconstruction.
2. Various surgical techniques have been cited within the collection of studies. Within the fusion group, both screw fixation and tightrope have been commonly mentioned and performed, whereas tendon transfer with an autograft is the more frequent method for reconstruction of AITFL.
3. AOFAS and OMA functional scores are most commonly used to evaluate progress post-operation within eighteen included studies. All return and follow-up patients demonstrate good to excellent functional scores after surgical intervention.
4. CT imaging evaluation has been reported in 12 studies which all of them deliver significant improvement post-operation.
5. No standardized surgical techniques or functional/imaging assessment has been determined
6. No significant complications from either technique

Syndesmosis injuries of the ankle joint can be confirmed by a collective of clinical parameters external rotation tests, and tenderness of anterolateral side test^30^ as well as radiological imaging stress X-ray, CT, MRI.^31^ Poor function scores and ankle instability is observed in non-operated conservative treatment.^32^ Overall operative outcomes are encouraging, however in regard to the main purpose of the narrative review; comparing and determining the superiority between fusion and reconstruction in improving functional syndesmosis instability, there is a lack of conclusive studies.

All studies, regardless of the surgical method, provide improved functional and radiological results. These studies’ respective focus are on; comparison between fixation methods (e.g. suture button vs screw),^7^^,^^9^^,^^14^^,^^16^^,^^25^ intra-patient group comparison, depending on the severity of injuries,^3^ outcomes of different grafts during reconstruction (research has shown positive reconstruction result using semitendinosus, peroneus longus)^1^^,^^4^^,^^20^^,^^24^ and non-comparative case/cohort studies.^5^^,^^17^, ^18^, ^19^^,^^21^, ^22^, ^23^ Colcuc et al.^4^ stated that the choice between fusion versus reconstruction can be determined by the degree and severity of the syndesmosis instability. Rammelt and Boszczyk suggested that acute or subacute syndesmosis injury can be treated by ankle arthroscopy with screw/suture button stabilization while chronic cases are managed by ligament reconstruction complemented by fusion/screw fixation.^33^ The above studies however lack a control group.

## Conclusion

All included studies demonstrate post-operative improvement, but the lack of comparative studies fails to determine the superiority of syndesmosis fusion against ligament reconstruction as the surgical intervention. Prospective and randomized trials comparing the clinical outcome of syndesmosis fusion versus ligament reconstruction would be required in the future to provide evidence for a more comprehensive analysis to identifying the most effective surgical intervention in treating syndesmotic injury.

## Funding

This research did not receive any specific grant from funding agencies in the public, commercial, or not-for-profit sectors.

## Authorship

Conception and design of study: SK Lim, YC Ho, SKK Ling.

Acquisition of data: SK Lim, YC Ho.

Analysis and/or interpretation of data: SK Lim, YC Ho.

Drafting the manuscript: SK Lim, YC Ho.

Revising the manuscript critically for important intellectual content: SKK Ling, PSH Yung.

Approval of the version of the manuscript to be published (the names of all authors must be listed): SK Lim, YC Ho, SKK Ling, PSH Yung.

## Declaration of competing interest

A conflict of interest occurs when an individual's objectivity is potentially compromised by a desire for financial gain, prominence, professional advancement or a successful outcome. AP-SMART Editors strive to ensure that what is published in the Journal is as balanced, objective and evidence-based as possible. Since it can be difficult to distinguish between an actual conflict of interest and a perceived conflict of interest, the Journal requires authors to disclose all and any potential conflicts of interest.

## List of abbreviations

AITFL: Anterior-Inferior Tibio-Fibular Ligament
PITFL: Posterior-Inferior Tibio-Fibular Ligament
IOL: interosseous ligament
PER: pronation-external rotation
SER: supination-external rotation
TCS: tibiofibular clear space
MCS: medial clear space
TFO: tibiofibular overlapping
CT: computer tomography
MRI: magnetic resonance imaging
PRISMA: Preferred Reporting Items for Systematic Reviews and Meta-Analyses
MINORS: Methodological Index for Nonrandomized Studies
AOFAS: American Orthopaedic Foot and Ankle Society
OMA: Olerud Molander Ankle Score
KAFS: Karlsson Ankle Functional Score
SD: standard deviation
